# Self-reported protein intake and properties of bone in community-dwelling older individuals

**DOI:** 10.1007/s11657-018-0421-0

**Published:** 2018-01-31

**Authors:** Jonas Johansson, Andreas Hult, Bente Morseth, Anna Nordström, Peter Nordström

**Affiliations:** 1 Department of Public Health and Clinical Medicine, Occupational and Environmental Medicine, Umeå University, Umeå, Sweden; 2 Department of Community Medicine and Rehabilitation, Geriatric Medicine, Umeå University, 90187 Umeå, Sweden; 3 Department of Community Medicine, The Arctic University of Norway, Tromsø, Norway; 4 School of Sport Sciences, The Arctic University of Norway, Tromsø, Norway

**Keywords:** Bone mineral density, Protein intake, Appendicular muscle mass, Community dwelling, Peripheral quantitative computed tomography

## Abstract

**Summary:**

This study revealed that a quick and simple estimation of protein intake was related to measures of bone density and area in 70-year-old individuals. Furthermore, these associations were mediated by muscle mass when investigating peripheral measurement sites such as arms and legs.

**Purpose:**

Recent evidence suggests that dietary protein is beneficial for bone health in older individuals, but less is known about the influence of muscle mass on this relationship. This cross-sectional study aimed to investigate associations among protein intake, bone health, and muscle mass in 2332 men and women aged 70 years.

**Methods:**

Volumetric bone mineral density of the radius and tibia was measured using peripheral quantitative computed tomography. Using dual-energy X-ray absorptiometry, we measured areal bone mineral density (aBMD) at the L1–L4 vertebrae, radius, and femoral neck, together with appendicular lean mass. Participants reported their average meal size and proportion of meat/fish intake. Associations were investigated using multiple linear regression models, adjusted for multiple covariates.

**Results:**

Self-reported protein intake was associated with aBMD of the femoral neck (*β* = 0.082) and L1–L4 vertebrae (*β* = 0.063) in men (both *p* < 0.05) after adjusting for multiple covariates, including appendicular muscle mass. No significant association was detected among women. In addition, protein intake was associated with tibial cortical area (*β* = 0.08), periosteal circumference (*β* = 0.072), radial aBMD (*β* = 0.064), and trabecular area (*β* = 0.078) in men (all *p* < 0.05), although these associations were attenuated after adjustment for appendicular muscle mass (all *p* > 0.05).

**Conclusion:**

Self-reported protein intake was associated with bone properties in 70-year-old men. The strength of these associations in peripheral bone sites may be partially mediated by muscle mass from protein intake.

## Introduction

Older individuals constitute a growing demographic proportion in the Western world, and improvements in lifestyle and functionality have become crucial factors for well-being and healthy aging [1]. Many diseases, especially osteoporosis, defined as the loss of bone mineral density (BMD) and disruption of bone microarchitecture, are associated with increasing age. Approximately one in five European women and one fifteenth of European men over the age of 50 years are affected, and the costs of orthopedic care and rehabilitation for osteoporotic patients are enormous; in Europe alone, they were estimated to total €37 billion in 2010 [2]. In addition, as much as 20% of 60–70 years old and nearly 50% of people older than 75 years suffer from sarcopenia, an age-related loss of muscle mass, strength, and functionality [3]. This condition is often a result of malnutrition and protein deficit and leads to poor musculoskeletal health [2–4].

Increasing dietary protein intake has been proposed as an approach to decrease musculoskeletal deficits in older individuals, and positive associations have been shown between higher dietary protein intake and lean body mass, indicating that sarcopenia and frailty can be prevented or postponed in these populations [5, 6]. In relation to bone health, protein supplementation has improved clinical outcomes in elderly patients with hip fractures, attenuating proximal femoral bone loss and shortening hospital stays [7].

Depending on dosage, dietary protein intake can be beneficial or detrimental to bone health [8, 9], although it is generally viewed positively as an influential factor in skeletal function and health. Several meta-analyses have shown positive associations of dietary protein intake with increased BMD of the lumbar spine [10, 11] and reduced risk of femoral neck fracture [12].

However, some aspects of these associations remain uncertain, such as the extent to which muscle mass can influence BMD variations in relation to protein intake among older individuals, and whether protein intake is related to BMD similarly at peripheral and central sites. With the present study, we aimed to explore associations among protein intake, bone properties, and appendicular lean mass in a cohort of community-dwelling older individuals using peripheral quantitative computed tomography (pQCT) and dual-energy X-ray absorptiometry (DXA).

## Material and methods

### Study population

**Fig. 1:**
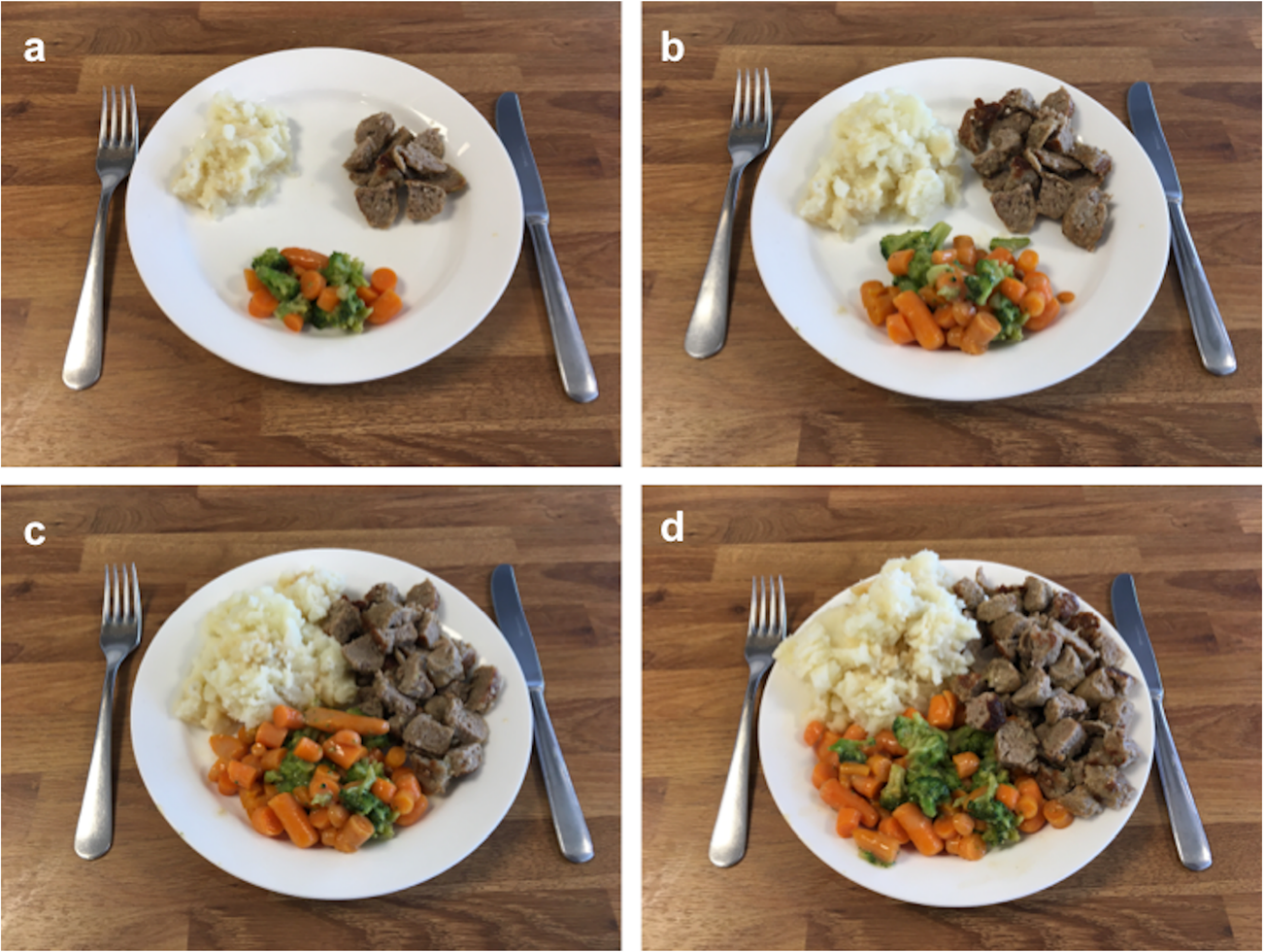
Photographs from the food questionnaire displaying amounts of meat/fish (top right part of the plate). Participants were asked to select the photograph that best represented their average meat/fish intake

This cross-sectional study is part of the Healthy Ageing Initiative (HAI), an ongoing cohort study conducted since June 2012 by researchers at Umeå University, Sweden. Details of the study population and procedure have been published elsewhere [13]; for more information, please visit www.healthyageinginitiative.com. Inclusion criteria for the HAI were residency in Umeå municipality and an age of 70 years at the time of participation. Eligible participants were drawn from population registers and invited via letter and subsequent telephone call. The sample for this study consisted of HAI participants for whom complete pQCT, DXA, accelerometer, and food questionnaire data were available (Fig. 1).

### Body composition and bone measurements

Lean body mass (in grams) and areal bone mineral density (aBMD, in grams per square centimeter) were assessed with a Lunar iDXA device (GE Healthcare Lunar, Madison, WI, USA). The appendicular lean mass index (iALM, in kilograms per square meter), an indicator of sarcopenia [14], was calculated as the sum of the lean mass of the arms and legs divided by height squared. aBMD was measured in the non-dominant radius, L1–L4 vertebrae, and femoral neck. For analysis, iDXA data were transformed from the natural units (grams per square centimeter) to milligrams per square centimeter to facilitate comparison with pQCT data.

Volumetric bone mineral density (vBMD, in milligrams per cubic centimeter) was measured with a pQCT device (XCT-2000; Stratec Medizintechnik, Pforzheim, Germany) in the non-dominant radius and tibia. The pQCT enabled analysis of more specific peripheral bone structures, such as by calculation of trabecular vBMD and cross-sectional area (CSA, in square millimeters), measured at 4% of total bone length in the distal–proximal direction. Cortical CSA, vBMD, peri- and endosteal circumferences (in millimeters), and cortical thickness (in millimeters) were measured at 66% of total bone length in the same direction. pQCT settings included a slice thickness of 2.0 mm and voxel size of 0.5 mm. Trabecular scan thresholds were set at 180 mg/cm^3^, and cortical scan thresholds were set at 280 mg/cm^3^. Experienced research nurses performed all measurements, and the iDXA and pQCT devices were calibrated each morning before measurement, according to standard procedure. Measurements were repeated if there were motion artifacts.

### Dietary, physical activity, and anthropometric assessments

Data on dietary habits were collected using a validated food frequency questionnaire [15], with estimation of meal size and the proportion of protein. Participants estimated the average proportion of protein in their meals with reference to four photographs depicting different amounts of meat/fish. Participants were instructed that this also included poultry and other sources of dietary protein, such as soy dairy. In a subsample analysis, 423 additional participants with the same inclusion criteria as the main cohort completed a previously validated 85-item food frequency questionnaire (FFQ) [16] that was later recalculated for total protein content in grams using a public nutrient content of food database available from the National Food Administration of Sweden (www.livsmedelsverket.se/en, accessed 2017-04-03). Physical activity (PA) was measured objectively with triaxial accelerometers (Actigraph, Pensacola, FL, USA). Collection, analysis, and interpretation of the accelerometer data have been described previously; in that study, these data showed associations between moderate to vigorous physical activity (MVPA) and bone parameters [13]. In accordance with these results, the average amount of moderate to vigorous physical activity (aMVPA) per day was used in the analysis for the current study. Accelerometer data were valid for the majority (92.9%) of participants, with wear times of at least 10 h/day for 4 days. Missing data were attributable to participants forgetting to wear the devices or to device malfunction. Standard anthropometric parameters, including height (in meters), weight (in kilograms), and waist and hip circumferences (in centimeters), were measured. The body mass index (BMI) was calculated as weight divided by height squared.

### Statistical analysis

The statistical analyses were performed using IBM SPSS Statistics 24.0 (IBM Corporation, Armonk, NY, USA). Values are presented as means ± standard deviations, except those for categorical variables, which are presented as percentages. Questionnaire-derived data on dietary and lifestyle variables were recoded using numeric scales for analysis. Differences between men and women were investigated using Student’s independent-samples *t* test for numerical variables and the chi-squared test for categorical variables. Potential associations between variables were investigated using Pearson’s bivariate correlations (*r*). Multiple linear regression models were used to adjust for covariates. Model 1 was unadjusted; model 2 was adjusted for sex, BMI, aMVPA/day, smoking, average meal size, and vegan/vegetarian diet; and model 3 was additionally adjusted for the iALM. All models were additionally stratified for women and men, with removal of the sex variable adjustment. Fully adjusted models were screened for variable influence factors in order to determine potential multicollinearity. Linear regression was also used in a sensitivity analysis to explore the relationship between estimated average protein content per serving and the amount of total protein (in grams) from the FFQ. For all analyses, a *p* level < 0.05 was deemed statistically significant.

### Ethical considerations

This study was approved by the Umeå University Research Ethics Committee (Dnr 07-031M) and conducted in line with the Helsinki Declaration of the World Medical Association. All participants provided written informed consent.

## Results

### Study cohort

The present study included 2332 HAI participants (50.1% men). The characteristics of the cohort are described in Table 1. Men had higher mean bone parameter, BMI, MVPA/day, and iALM values than did women (all *p* < 0.001). Men also reported larger meal sizes and greater meat/fish intake than did women (both *p* < 0.001). The cohort contained more female than male smokers and vegetarians/vegans, although these differences were not statistically significant. Additionally, validation of estimated meat/fish intake was done in a subsample of 423 individuals who completed an 85-item food frequency questionnaire (FFQ). In relation to protein content arrived from the FFQ, we found that the mean protein intake was 62.5 ± 20.4 and 71.1 ± 21.4 g of protein per day for women and men, respectively. Every increase in meal size of meat/fish was also associated with an increase of 8.94 (95% CI 4.27–13.60) grams of protein for women and 9.14 (95% CI 4.88–13.40) grams of protein for men. This association was significantly correlated (standardized *β* 0.247 for women and 0.286 for men, *p* < 0.001 for both).

**Table 1:** Characteristics of the study cohort

Variable	All (*n* = 2332)	Women (*n* = 1164)	Men (*n* = 1168)	*p*
Height (m)	1.70 ± 0.09	1.63 ± 0.06	1.77 ± 0.06	**
Weight (kg)	76.43 ± 13.94	69.55 ± 12.33	83.29 ± 11.93	**
BMI (kg/m^2^)	26.35 ± 4.01	26.02 ± 4.39	26.69 ± 3.57	**
Appendicular lean mass index (kg/m^2^)	7.12 ± 1.11	6.37 ± 0.80	7.88 ± 0.84	**
Appendicular lean mass (kg)	20.81 ± 4.68	17.05 ± 2.43	24.60 ± 3.08	**
Arm lean mass index (kg/m^2^)	1.80 ± 0.39	1.50 ± 0.22	2.10 ± 0.28	**
Leg lean mass index (kg/m^2^)	5.33 ± 0.77	4.88 ± 0.63	5.78 ± 0.62	**
Smoker (%)	5.8	6.7	4.9	
Vegetarian/vegan diet (%)	0.3	0.3	0.2	
Average MVPA/day (min)	33.18 ± 25.41	30.93 ± 23.50	35.46 ± 27.03	**
Estimated average meal size (%)				ǂ
< 1/2 portion	0.3	13.7	10.6	
1/2 portion	4.0	6.4	1.6	
3/4 portion	12.1	0.3	0.3	
Normal portion	75.9	74.8	77.1	
Large portion	7.6	4.8	10.4	
Estimated average portion of meat/fish (%)^a^			ǂ	
A (< 1/2 portion)	7.4	10.9	3.9	
B (3/4 portion)	61.7	69.2	54.2	
C (normal portion)	28.4	18.8	37.9	
D (large portion)	2.5	1.0	4.0	
DXA				
Femoral neck aBMD (mg/cm^2^)	869.76 ± 134.56	815.35 ± 111.84	923.96 ± 133.44	**
L1–L4 vertebral aBMD (mg/cm^2^)	1146.33 ± 215.20	1049.95 ± 180.43	1243.30 ± 203.54	**
Radius 33% aBMD (mg/cm^2^)	843.60 ± 160.61	719.44 ± 108.91	967.76 ± 94.32	**
pQCT				
Radius				
Trabecular area (mm^2^)	198.78 ± 40.54	170.82 ± 24.13	226.62 ± 33.88	**
Trabecular vBMD (mg/cm^3^)	180.61 ± 44.42	156.83 ± 37.59	204.28 ± 37.53	**
Cortical area (mm^2^)	81.74 ± 24.88	61.78 ± 12.94	101.62 ± 16.64	**
Cortical vBMD (mg/cm^3^)	1105.2 ± 54.16	1091.56 ± 50.01	1118.77 ± 54.73	**
Cortical thickness (mm)	2.16 ± 0.57	1.77 ± 0.40	2.55 ± 0.44	**
Periosteal circumference (mm)	44.48 ± 5.51	40.81 ± 3.63	48.14 ± 4.54	**
Endosteal circumference (mm)	30.90 ± 5.18	29.68 ± 4.62	32.12 ± 5.42	**
Tibia				
Trabecular area (mm^2^)	534.71 ± 97.39	477.84 ± 77.75	591.40 ± 80.48	***
Trabecular vBMD (mg/cm^3^)	218.95 ± 41.75	203.11 ± 40.51	234.74 ± 36.70	**
Cortical area (mm^2^)	295.83 ± 71.02	242.19 ± 41.86	349.28 ± 50.99	**
Cortical vBMD (mg/cm^3^)	1089.52 ± 38.42	1078.89 ± 40.34	1100.12 ± 33.19	**
Cortical thickness (mm)	3.72 ± 0.82	3.25 ± 0.67	4.20 ± 0.66	**
Periosteal circumference (mm)	91.18 ± 9.18	85.61 ± 6.67	96.74 ± 7.89	**
Endosteal circumference (mm)	67.78 ± 9.32	65.22 ± 8.79	70.34 ± 9.13	***

Values are presented as means ± standard deviations, except where otherwise indicated

*BMI* body mass index, *MVPA* moderate to vigorous physical activity, *DXA* dual-energy X-ray absorptiometry, *aBMD* areal bone mineral density, *pQCT* peripheral quantitative computed tomography, *vBMD* volumetric bone mineral density

**p < 0.01; ***p < 0.001, men vs. women (independent-samples *t* test); ǂ*p* < 0.001, men vs. women (Pearson chi-squared test)

^a^Photographs are provided in Fig. 1

### Associations between estimated protein intake and bone parameters

In the unadjusted model 1, all bone parameters investigated by DXA and pQCT were associated significantly with estimated protein intake in the total cohort (Table 2). These associations were attenuated when data from men and women were analyzed separately, and they were generally stronger among men than among women. In model 2, adjusted for BMI, average meal size, vegan/vegetarian diet, smoking, and aMVPA/day, associations between estimated protein intake and bone parameters were not significant among women; among men, the strongest association was with femoral neck aBMD (*β* = 0.086, *p* < 0.01). Significant associations were also found among men between protein intake and L1–L4 vertebral aBMD (*β* = 0.066), radial aBMD (*β* = 0.064), tibial cortical area (*β* = 0.08), tibial periosteal circumference (*β* = 0.072), and radial trabecular area (*β* = 0.078; all *p* < 0.05; Table 2). In the final linear regression model (model 3), additionally adjusted for the iALM, protein intake remained unassociated with any bone parameter among women (Table 2). Positive associations with femoral neck aBMD (*β* = 0.082, *p* < 0.05) and L1–L4 vertebral aBMD (*β* = 0.063, *p* < 0.05) persisted among men. However, no further significant association was observed between protein intake and properties of peripheral bone sites (measured by pQCT) or radial aBMD (measured by DXA). Additionally, adjusting for body fat percentage instead of BMI in model 3 for central bone sites did not affect the associations (L1–L4 vertebral aBMD: *β* = 0.065 and femoral neck aBMD: *β* = 0.083, *p* < 0.05 for all, data not presented in Table 2). Finally, there was a significant correlation (*p* ≤ 0.001 for all) between protein intake and iALM in the total cohort (*r* = 0.25), and for men (*r* = 0.12) and women (*r* = 0.10) separately.

**Table 2:** Results of linear regression analysis of the associations between estimated protein intake and bone properties

Variable	Model 1			Model 2			Model 3		
	All	Women	Men	All	Women	Men	All	Women	Men
DXA									
L1–L4 vertebral aBMD	0.138***	−0.009	0.056	0.018	−0.035	0.066*	0.014	−0.040	0.063*
Femoral neck aBMD	0.155***	0.036	0.076*	0.044*	0.002	0.086**	0.042*	0.001	0.082*
Radius 33% aBMD	0.237***	0.05	0.064*	0.029*	0.023	0.064*	0.025	0.019	0.052
pQCT									
Tibia									
Trabecular area	0.194***	0.065*	0.049	0.031	0.05	0.028	0.015	0.04	0.001
Trabecular vBMD	0.119***	0.017	0.032	0.021	−0.007	0.055	0.022	−0.008	0.056
Cortical area	0.234***	0.046	0.077**	0.038*	0.02	0.08*	0.028	0.013	0.060
Cortical vBMD	0.077***	0.028	−0.017	0.015	0.024	0.016	0.022	0.031	0.021
Cortical thickness	0.174***	0.022	0.036	0.022	0.005	0.045	0.02	0.006	0.039
Periosteal circumference	0.207***	0.054	0.077**	0.05**	0.041	0.072*	0.033	0.023	0.049
Endosteal circumference	0.109***	0.031	0.05	0.037	0.029	0.04	0.022	0.015	0.025
Radius									
Trabecular area	0.216***	0.016	0.081**	0.036*	0.006	0.078*	0.02	−0.007	0.051
Trabecular vBMD	0.136***	−0.005	0.008	0.009	−0.008	0.02	0.011	−0.008	0.024
Cortical area	0.245***	0.062*	0.07*	0.038**	0.051	0.062	0.026	0.040	0.034
Cortical vBMD	0.100***	0.021	0.035	0.037	0.028	0.042	0.027	0.029	0.021
Cortical thickness	0.211***	0.045	0.055	0.037*	0.043	0.044	0.031	0.039	0.023
Periosteal circumference	0.209***	0.052	0.045	0.029	0.03	0.045	0.008	0.014	0.008
Endosteal circumference	0.073***	0.017	0.008	0.005	0.001	0.014	−0.013	−0.01	−0.010

Values are presented as standardized *β* coefficients. Model 1 was unadjusted. Model 2 was adjusted for body mass index, estimated average meal size, average amount of moderate to vigorous physical activity per day, smoking, and diet. Model 3 was additionally adjusted for the appendicular lean mass index. All models were stratified for women and men and were thus automatically adjusted for sex

*DXA* dual-energy X-ray absorptiometry, *aBMD* areal bone mineral density, *pQCT* peripheral quantitative computed tomography, *vBMD* volumetric bone mineral density

*p < 0.05; **p < 0.01; ***p < 0.001

## Discussion

The current study explored the relationships between protein intake and bone properties with adjustment for known and potential confounders. Our findings suggest that self-assessed protein intake is associated independently with bone parameters (measured by iDXA and pQCT) in men, but not in women. Furthermore, they suggest that the associations between bone properties at peripheral sites and protein intake are mediated by appendicular muscle mass.

The link between protein intake, bone properties as measured by DXA, and reduced fracture risk has been suggested previously [17]. Several meta-analyses have also shown positive associations between protein intake, spinal BMD and reduced hip fracture risk [10–12]. This study confirms these findings and expands upon the knowledge by also providing pQCT data and “true” volumetric BMD in older individuals. Furthermore, the novelty of the current findings pertains to the mediating effect of appendicular muscle mass on bone properties at peripheral sites, such as the tibia and radius. Protein supplementation has been associated with increased serum levels of insulin-like growth factor-1 (IGF-1), which is anabolic for the musculoskeletal apparatus. Thus, the positive association between protein intake, lean body mass, and bone properties may to some degree be explained by higher levels of IGF-1, as also indicated by previous research [7, 18]. A previous study revealed significant associations between peripheral bone parameters and appendicular muscle mass, independent of PA, which further supports a muscle–bone relationship, although no adjustment for dietary protein intake was made [19]. In contrast to peripheral bone sites, the association between dietary protein content and central bone sites prevailed despite adjustments for muscle mass. This held true even after BMI was substituted in the fully adjusted model, suggesting that the association between dietary protein intake and central bone properties is not mediated by an energy surplus, resulting in increased body fat.

An interesting, but perhaps discouraging finding was the lack of association between protein intake and bone parameters in women, who experience an increased prevalence of osteoporosis at older ages than do men [20]. Studies have suggested that females tend to underestimate their performance on self-evaluation questionnaires compared with males [21], which could partly explain the sex discrepancy, assuming that this trend also applies to self-reported protein intake. However, the regression models were adjusted for average meal size, and the probability that only women underestimated their protein intake in relation to average meal size seems to be low. Furthermore, women in this age group have undergone menopause, and thus have already experienced the associated decline in BMD [22]. Men, on the other hand, tend to lose radial and hip BMD in a more linear pattern after the age of 50 years [23]. The substantial menopause-associated decline in estrogen levels among females could also partly explain the sex discrepancy in our results. Although many studies have investigated bone health and diet in postmenopausal women, they have produced differing results [24]; thus, consensus on this point cannot be established.

This study has some limitations to consider. First, the cross-sectional design prevented us from investigating causal effects of the relationships between protein intake and bone parameters. Second, the inclusion of participants at the exact age of 70 years limits generalizability to other elderly populations, although the analysis of this cohort was thus automatically age adjusted. Third, our estimations of protein intake were based on assessment of meat/fish consumption. As such, there is a possibility that other nutrients, which we have not controlled for, might influence the associations. Fourth, one possible source of bias is confounding by variables for which we did not control, such as socioeconomic status, resistance exercise, and vitamin D status. Five, the dietary questions used in the current study may seem to be simplistic, as participants chose one of four photographs that best represented their average protein intake. However, the results suggest that significant associations can be detected with such simple measures. Study participants, especially those who are elderly, often have difficulty reliably estimating the amounts of different dietary components using comprehensive questionnaires [25]. In addition, the use of more precise dietary assessment tools, such as 24-h recall measures, is more time consuming, requires the involvement of trained dieticians, and relies heavily on participants’ ability to recall and summarize their dietary habits. Thus, visual representations of average dietary intake could be a viable alternative to more comprehensive methods, especially since we found that meal size estimation of protein content correlated quite well with total protein intake in grams from the FFQ in the sensitivity analysis.

This study also includes several strengths. The cohort size gives the results a certain power and the ethnic homogeneity of the study group enables the generalization of the results to elderly Caucasian populations. We were able to adjust for average meal size in the regression analysis, providing more reliable data on the specific contribution of protein intake to bone parameters. The use of objective measures of PA also increased the accuracy of the regression results, as studies have revealed difficulties with the subjective estimation of PA [26, 27]. Furthermore, the availability of DXA and pQCT data from multiple sites with different bone properties, compared with DXA data alone, enabled us to investigate peripheral bone sites and the potential mediating effect of appendicular lean mass on bone properties. Most previous researchers utilized DXA to determine aBMD, although this technique involves the two-dimensional measurement of aBMD and may produce measurement errors [28, 29]. The advantage of using pQCT is that it enables the three-dimensional characterization of trabecular and cortical aspects of bone structures, thereby providing “true” vBMD data [30].

In conclusion, self-reported protein intake, acquired using a quick and simple method, was associated with bone properties in 70-year-old men, but not in women of the same age. Associations of protein intake with peripheral bone parameters, such as those of the radius and tibia, may be mediated by appendicular muscle mass. Further studies, especially randomized controlled trials, are needed to investigate the effects of dietary protein on bone properties and muscle mass in elderly individuals.
